# Informatics methods for laboratory evaluation of HPV ordering patterns with an example from a nationwide sample in the United States, 2003-2009

**DOI:** 10.4103/2153-3539.73504

**Published:** 2010-12-03

**Authors:** Brian H. Shirts, Brian R. Jackson

**Affiliations:** 1 Department of Pathology, University of Utah School of Medicine, Salt Lake City, UT; 2 ARUP Institute for Clinical and Experimental Pathology, Salt Lake City, UT

**Keywords:** Cancer prevention, cervical cancer, clinical laboratory informatics, Human papillomavirus, test utilization

## Abstract

**Background:**

Laboratory data is a rich source of information that can be used to estimate adherence to physician guidelines and motivate improvement in clinical practice. Human papillomavirus (HPV) testing is an important component of cervical cancer screening programs with established screening guidelines. The purpose of this study was to develop methods to estimate concordance with published guidelines for HPV testing in order to provide clinicians and payors specific feedback about overscreening.

**Methods:**

This retrospective analysis of laboratory test ordering patterns evaluated 454,532 HPV tests ordered from September 2003 to October 2009 from 110 facilities and performed at ARUP laboratories. We used laboratory data including patient demographics, ordering frequency, timestamps and results to examine the proportion of HPV tests ordered on women under 21 years, ordered on women between 21 and 29 years apparently before cytological examination, repeated less than 1 year after a positive HPV result in women over 30 years, and repeated less than 3 years after a negative HPV result in women over 30 years.

**Results:**

The absolute number and proportion of HPV tests performed on women under 21 years declined from 20% in 2005 to 5% in October 2009. The proportion of HPV tests performed women between 21 and 29 years also declined during this period. Approximately one-third of HPV tests performed on women between 21 and 29 years arrived for HPV testing before cervical screening had presumably been completed. The most common follow-up intervals for HPV testing on women over 30 years were 6 months following a positive HPV result and 12 months following a negative HPV result. Only 6% of repeat HPV testing in women over 30 years followed a negative HPV result by 3 years or more. Approximately one-fourth of HPV tests ordered the year ending October 2009 were unnecessary based on the American Society for Colposcopy and Cervical Pathology guideline.

**Conclusions:**

We demonstrate simple methods to evaluate appropriate utilization of HPV testing using laboratory data. Our data illustrates that some aspects of HPV test ordering have become more consistent with guidelines over time. However, a large portion of HPV testing in the United States is unnecessary. This highlights opportunities for optimization of a rational cancer prevention strategy to reduce unnecessary screening, colposcopy and biopsies.

## INTRODUCTION

As the US healthcare system moves toward greater promotion of evidence-based practice, it is critical to develop better measurement systems to evaluate concordance between practice and evidence. Laboratory data is one of the richest sources of such information. This paper illustrates how a laboratory can take advantage of multiple attributes from laboratory data, including patient demographics, ordering frequency, timestamps (i.e. database records of when samples are collected and arrive in lab), and results to estimate adherence to an evidence-based guideline.

Testing for human papillomavirus (HPV) is a widely used component of cervical cancer screening programs, and may become more important as studies continue to support its effectiveness.[1] In the US most primary care clinicians report cervical cancer screening guidelines being very influential in their practice, yet surveys and observational data indicate that overscreening may be common.[2,3] HPV testing is a relatively new component of cervical cancer screening protocols and its utilization has not been extensively studied. Clinical laboratories have the opportunity to improve the quality of medical care by providing ordering clinicians feedback about overuse of HPV testing.

We analyzed HPV test ordering patterns at a US national referral laboratory to estimate concordance with published guidelines. The American Society for Colposcopy and Cervical Pathology (ASCCP) has published a widely cited guideline for cervical cancer screening which has been reiterated by other professional organizations including the American College of Obstetricians and Gynecologists and the American Society for Clinical Pathology.[4–8] These guidelines state that HPV testing is contraindicated in women under 21 years, and that even if HPV testing is performed on women under 21 years HPV results should not influence clinical decisions. In women between 21 and 30 years, HPV testing should not be used in primary screening, but may be used for evaluating certain cervical lesions identified by cytology. In women 30 years and over, HPV testing may be used for both evaluating cervical lesions and for screening. In women over 30 years with negative screening HPV and negative cytology, annual screening is not indicated and the interval for follow-up screening may be safely extended to 3 years,[4,5] These replaced the earlier ASCCP guideline, which recommended HPV screening for stratification of certain cervical lesions without reference to age.[9]

The ASCCP guideline identifies two specific situations in which HPV testing should be repeated in less than 3 years.[5] In patients with atypical squamous cells of undetermined significance (ASC-US) and positive HPV results but negative colposcopy findings, HPV should be repeated in 1 year; but shorter intervals are not indicated. In the rare situation when atypical glandular cells are detected, HPV testing is indicated to stratify lesions with repeat HPV testing suggested 6 months after a positive HPV result and 12 months after a negative HPV result.

In order to evaluate physician compliance with the ASCCP guideline with regard to HPV testing, we used laboratory information to calculate four measures: 1) Proportion of HPV tests ordered on women under 21 years; 2) Proportion of women between 21 and 29 years for whom HPV tests appear to have been ordered before cytological examination; 3) Proportion of repeat HPV tests performed less than 1 year after a positive HPV result in women over 30 years; 4) Proportion of repeat HPV tests performed less than 3 years after a negative HPV result in women over 30. We then show how these values can be combined to estimate the total proportion of unnecessary HPV tests ordered at an individual laboratory.

## METHODS

ARUP, a national reference laboratory affiliated with the University of Utah Department of Pathology, provides HPV testing for several hundred hospitals and regional laboratories across the US. As a component of our laboratory service we provide analyses of test ordering practices, in which we identify potential overuse and underuse of laboratory tests in order to help health care facilities improve the quality and lower the cost of their services. In order to further develop our analyses of HPV ordering practices we evaluated data from 454,532 HPV tests ordered September 2003, when HPV testing was initiated at ARUP, to October 2009 at 110 facilities that each averaged over 20 HPV test orders per month during the study period. We limited evaluation to facilities which averaged over 20 HPV tests per month as to evaluate only facilities which perform routine HPV testing at ARUP and to exclude facilities that only send quality control or confirmation samples to ARUP. Data was stored as discrete data elements in a translational SQL database, retrieved using Crystal Reports v11.5 (SAP Business Objects, Waldorf, Germany), and validated with queries using SQL Server 2008 Management Studio v10.0 (Microsoft, Seattle, WA). All analysis was performed using Microsoft Excel 2007 (Microsoft).

We calculated the proportion of HPV tests performed on women under 21 years and under 30 years during the study period. To estimate the proportion of women of 21-29 years for whom cytology had been performed before HPV testing we evaluated data from selected months, May 2005, May 2007 and May 2009. Results were similar for each of these 3 months, so we did not evaluate this measure the entire study period. We used a comparison of time from sample collection to time of sample arrival at the laboratory with the median turnaround time for cytological examination at the University of Utah, Department of Pathology, which was approximately 7 days, to estimate the proportion of tests that had undergone previous cytological review. Discussions with cytologists revealed that it would be highly unlikely for an abnormal sample to be collected, sent to a pathology laboratory for review, reviewed by a cytology technician, reviewed by a pathologist and referred to our laboratory in less than 5 days. Thus, time from collection to time of arrival in lab can serve as an easily accessible surrogate that could obviate the need for detailed review of cytology records to determine the appropriateness of test orders on women between 21 and 29 years.

To study follow-up times, we identified 12,523 HPV tests on patients who were over 30 years at the time of initial HPV testing and for whom repeat testing had been performed during the 1-year period between November 2008 and October 2009. Although we limited our evaluation to testing repeated the last year of the study, initial testing, which determined the appropriateness of the repeat test, was performed any time between September 2003 and the end of the study. We separated these paired tests into groups based on the outcome of the initial HPV test.

**Figure 1:**
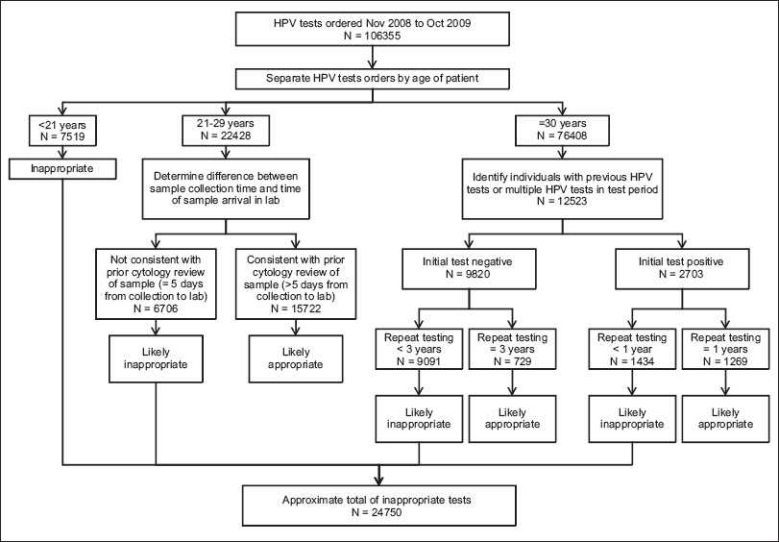
Workflow diagram illustrating data used in evaluation of proportion of unnecessary tests

We used the results of each of these analyses to identify the number of apparently inappropriate tests performed for between November 2008 and October 2009 [Figure 1]. We combined this information from with information about average frequencies of abnormal findings in cervical cytology[10] to estimate the total percentage of unnecessary tests ordered during 1 calendar year.

This project was approved by the University of Utah IRB_00039168.

## RESULTS

*Proportion of HPV tests ordered on women less than 21 years*.

**Figure 2:**
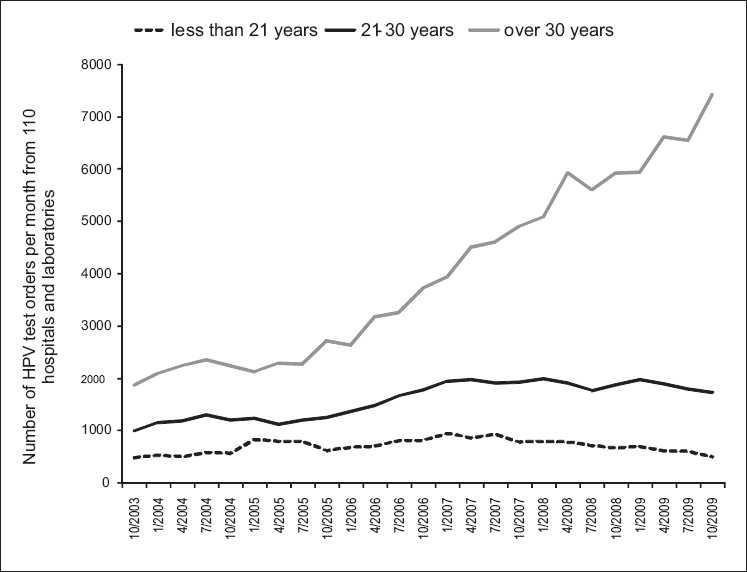
Trends in HPV ordering by age of patient October 2003-October 2009

**Figure 3:**
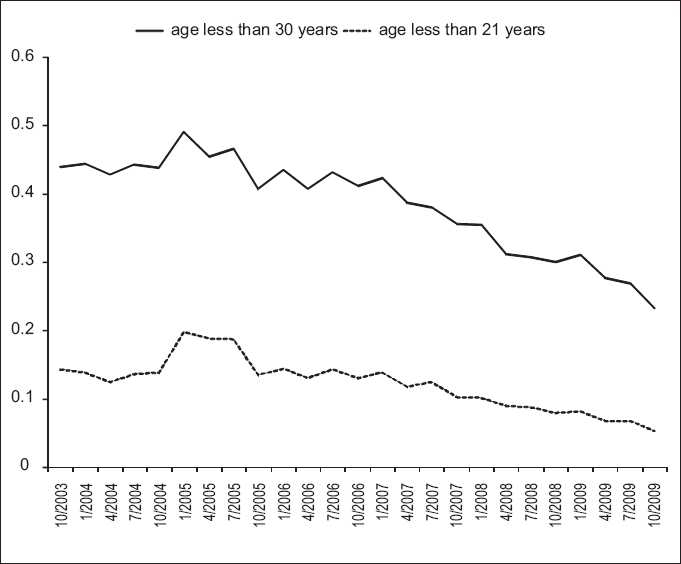
Trends in HPV ordering October 2003-October 2009, proportion of ordered tests on young women. Note the top line charts the prortion of all women under 30 (IE under 21 and 21-29 groups combined)

There was both an absolute and relative reduction in testing on women under 21 years and increased testing on women over 30 years [Figures 2 and 3]. The proportion of HPV tests ordered for women less than 21 years declined since early 2005 from a high of 20% in 2004 to 5% in October 2009 [Figure 3].

Proportion of women between 21 and 29 years for whom HPV tests were presumably ordered before cytological examination.

Although the absolute number of HPV tests ordered for women of age 21-29 years, the proportion of HPV tests ordered for women of age 21-29 years declined from 30% in 2004 to 18% in October 2009 [Figures 2 and 3]. The distribution of the difference from sample collection time to sample arrival at the laboratory during May 2005, May 2007 and May 2009 was similar, showing a bimodal distribution with a narrow, positively skewed peak at 2 days; a broader, similarly skewed peak at 8 days; and a trough at 6 days. This distribution is consistent with some samples being processed immediately and sent to the referral laboratory for HPV testing (peak at 2 days) and others being evaluated for cytology before being sent for HPV testing (peak at 8 days). Thirty-three percent, 32% and 31% of HPV tests performed on women between 21 and 29 years arrived at the laboratory 5 or fewer days after collection for May of 2005, 2007 and 2009, respectively.

Proportion of repeat HPV tests performed less than 1 year after a positive HPV result in women over 30 years

**Figure 4:**
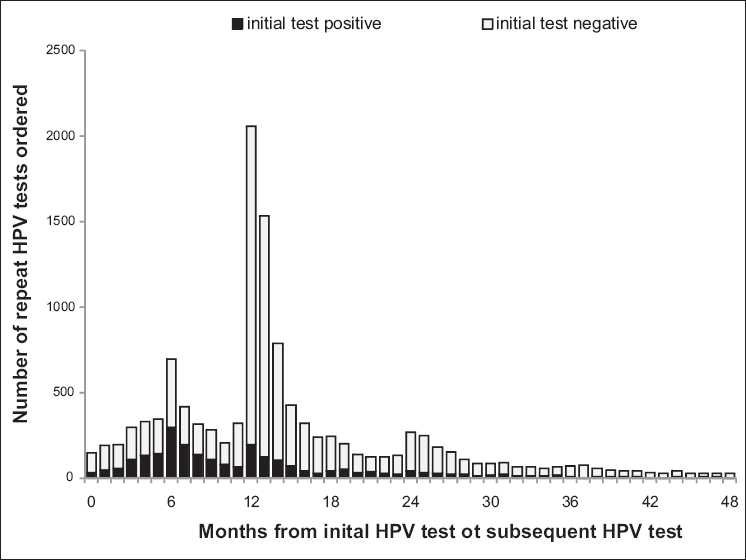
Distribution of time between HPV tests on women over 30

Between November 2008 and October 2009 the most common observed follow-up intervals for HPV testing on women over 30 years following a positive HPV test result was 6 months [Figure 4]. For these patients who were initially HPV-positive, 66% were still positive when tests were repeated after 6 months and 59% were positive when tests were repeated at 12 months.

Proportion of repeat HPV tests performed less than 3 years after a negative HPV result in women over 30 years.

The most common observed follow up intervals for HPV testing on women over 30 years after a negative test HPV results was 12 months [Figure 4]. For these patients who were initially HPV negative, 7% were HPV positive when tests were repeated after 6 months and 4% were positive when tests were repeated at 12 months. Only 6% of repeat HPV testing in women over 30 years followed a negative HPV result by 3 years or more [Figure 4].

*Total proportion of unnecessary HPV tests during 1 calendar year*.

Between November 2008 and October 2009 7.1% of all HPV tests were ordered on women less than 21 years, all of which were inappropriate and unnecessary according to ASCCP guideline [Figure 1]. During this time period 21% of all HPV tests were ordered on women between 21 and 29 years; 30% of these did not arrive in the laboratory long enough after collection for previous cytological screening, suggesting an additional 6.3% (30% of 21%) of total ordered HPV tests were inappropriately premature. Approximately 5% would presumably have been positive for ASC-US during cytology screening,[10] so 6% (95% of 6.3%) of ordered tests would have been not only inappropriately premature but unnecessary per ASCCP guideline. Repeat screening of previously HPV positive women less than 12 months after previous screening represented 1.3% of all HPV test orders. It is unlikely that more than a quarter of this testing was performed on atypical glandular cells,[10] suggesting an additional 1% of HPV tests were inappropriately ordered, although inappropriately premature, many of these tests should have been performed after 1 year, so should not be considered completely unnecessary. Finally, 8.5% of all HPV tests ordered were on women over 30 years who had a previously negative HPV test at the same facility within the previous 3 years. HPV positivity rates suggest that almost all of these tests were on low-risk women and were inappropriate and unnecessary per ASCCP guideline. In total, approximately 23% of HPV tests ordered between November 2008 and October 2009 were inappropriate and approximately 22% were unnecessary.

## DISCUSSION

The proportion and absolute number of young women receiving unnecessary HPV testing has decreased since 2005. Likewise, the proportion of women 21-29 years receiving HPV testing decreased since 2005, but the absolute number of tests on this population did not change. Throughout the study period approximately one-third of HPV tests performed on women between 21 and 29 years arrived at the laboratory less than 5 days after collection, suggesting screening rather than follow-up evaluation of abnormal cytology. Retesting for HPV was performed more frequently than expected, with most tests performed 6 months after positive and 12 months after negative HPV results, even though clinical indications for these screening intervals are very rare.[10] HPV positivity rates 12 months after negative tests were similar to those reported in population screening[11] suggesting most of these were simply repeat tests in low-risk women as opposed to stratification of repeated ASC-US findings on cytology. Initial guidance for using negative HPV screening to identify low-risk women and extend screening intervals was published 5 years before our study ended,[4] and guidelines to screen low-risk women triennially have been periodically repeated for over two decades.[12–14] Yet use of HPV testing to support triennial screening appears uncommon. These findings are consistent with studies of traditional cervical screening which have shown reluctance to move away from annual screening.[3] However, increased volumes of HPV tests in women over 30 years may foreshadow an increase in triennial cervical cancer screening.

The accuracy of our retrospective evaluation of test ordering patterns may be limited by lack of both cytology results and clinical information about the patients on whom these tests were ordered. However, clinical laboratories that wish to evaluate overuse of HPV testing for their clients are unlikely to have the resources to perform detailed reviews of cytology and clinical information to determine the appropriateness of tests. Our methods illustrate rapid, automatable methods that we believe produce results sufficiently accurate to indicate where improvement in clinical practice is necessary and possible.

Previous studies evaluating utilization of cervical cancer screening have used surveys or billing data to evaluate utilization of cervical cancer screening.[2,15] Surveys are subject to bias as clinicians are likely to report better adherence to guidelines than actual practice, patients may recall cervical screening but not be aware of the exact tests ordered, and billing data usually does not contain information about test results or transport times. Our methods based on test orders and HPV test results from a large, representative nationwide sample of regional laboratories and hospitals are not subject to these biases. As such, we believe that these results represent a broad estimate of HPV overuse in the US and that other clinical laboratories that perform HPV testing may find similar results, and should be able to use these results as benchmarks.

Combining data from all of our endpoints, we estimate that approximately one-fourth of HPV tests ordered in the US in the year ending October 2009 were unnecessary based on the ASCCP guideline. Overuse of HPV tests does not have a place in a rational cancer prevention strategy and leads to additional clinical and financial costs in the form of unnecessary follow-up screening, colposcopy and biopsies. Our data highlights opportunities for laboratories to use informatics tools in the optimization of a rational cancer prevention strategy in the US.
